# Draft Genome Sequence of *Mariprofundus ferrooxydans* Strain JV-1, Isolated from Loihi Seamount, Hawaii

**DOI:** 10.1128/genomeA.01118-15

**Published:** 2015-10-08

**Authors:** Heather Fullerton, Kevin W. Hager, Craig L. Moyer

**Affiliations:** Department of Biology, Western Washington University, Bellingham, Washington, USA

## Abstract

*Mariprofundus ferrooxydans* strain JV-1 was isolated in 1998 from Loihi Seamount, Hawaii. Here, we present the draft genome of strain JV-1, which shows similarity to other sequenced *Mariprofundus* isolates, strains PV-1 and M34.

## GENOME ANNOUNCEMENT

*Mariprofundus ferrooxydans* strain PV-1 is the type strain of the class *Zetaproteobacteria.* These organisms are classified as marine neutrophilic iron-oxidizers and were first identified from Loihi Seamount, Hawaii (1). This taxon was originally identified as a deeply rooted *Gammaproteobacteria* based on SSU rRNA sequence identity alone (2). Further sequence analysis of multiple genes from *M. ferrooxydans* PV-1 placed these organisms in a novel class (1). *Zetaproteobacteria* have been found throughout the Pacific and Atlantic Oceans at hydrothermal sites with elevated levels of reduced iron in the vent effluent (3).

We examined the genetic features of the *M. ferrooxydans* strain JV-1 through whole-genome sequencing. This is the third sequenced isolate to date of the class *Zetaproteobacteria*. Source microbial mat material for isolation of strain JV-1 was from Loihi Seamount, Lower Jet Vents near Marker 11, collected in October of 1998 at a depth of 1,298 m, inside the Pele’s Pit Caldera using submersible *Pisces V* (Dive No. 393). An axenic culture was prepared by dilution to extinction series in FeS gradient tubes as previously described (4). A frozen cell suspension of *M. ferrooxydans* JV-1 (ATCC BAA-1021) was grown was grown as recommended in artificial salt-water gradient plates with FeS as the bottom layer. DNA was extracted from cell pellets using the FastDNA Spin Kit for Soil (MP Biomedicals, Solon, OH). Extracted DNA was cleaned and concentrated with an Aurora instrument (Boreal Genomics, Vancouver, BC, Canada). A sequencing library was prepared with the Illumina Nextera DNA sample prep kit and subsequently sequenced with MiSeq reagent kit v3 (600 cycle) using 300 bp paired-end reads on an Illumina MiSeq platform (Illumina, San Diego, CA). A total of 17.6 million reads were produced yielding 7.5 Gb of data. Read quality was assessed with FastQC (http://www.bioinformatics.bbsrc.ac.uk/projects/fastqc/). Quality controlled reads were used to assemble the draft genome with SPAdes v3.5.0 (5). This resulted in 48 contigs with an *N*_50_ of 130,211 bp and the largest contig is 205,763 bp. This assembly of the draft genome is 2,856,365 bp with a G+C content of 53.95%.

The draft genome was annotated by the NCBI Prokaryotic Genome Annotation Pipeline (PGAP) (http://www.ncbi.nlm.nih.gov/genome/annotation_prok/). From this annotation, we identified 2,627 coding sequences (CDs), five rRNAs, and 46 tRNAs. The SSU rRNA and LSU rRNA genes were encoded on a single contig with the tRNAs for alanine and isoleucine between the two rRNA genes. This is the same arrangement of genes as in *M. ferrooxydans* PV-1. Average nucleotide identity comparison to other *M. ferrooxydans* PV-1 and M34 genomes showed a 99.89% and 97.8% identity, respectively. This analysis places JV-1 as a member of *Zetaproteobacteria* OTU-11 along with strains PV-1 and M34 (6). Like PV-1 and M34, strain JV-1 encodes genes for growth by chemoautotrophy and motility via a flagellum.

### Nucleotide sequence accession number.

The completed genome sequence of *M. ferrooxydans* JV-1 has been deposited in the GenBank database under the accession number LIGC00000000. The version described in this paper is the first version.
